# The impact of HIV status on the distance traveled to health facilities and adherence to care. A record-linkage study from rural South Africa

**DOI:** 10.7189/jogh.10.020435

**Published:** 2020-12

**Authors:** Paul Mee, Brian Rice, Chodziwadziwa Whiteson Kabudula, Stephen M Tollman, Francesc Xavier Gómez-Olivé, Georges Reniers

**Affiliations:** 1 Department of Infectious Disease Epidemiology, Faculty of Epidemiology and Public Health, London School of Hygiene and Tropical Medicine, London, UK; 2 The MeSH Consortium, London School of Hygiene and Tropical Medicine, London, UK; 3 Medical Research Council/Wits University Rural Public Health and Health Transitions Research Unit (Agincourt), School of Public Health, Faculty of Health Sciences, University of the Witwatersrand, Johannesburg, South Africa; 4 Department of Public Health, Environments and Society, Faculty of Public Health and Policy, London School of Hygiene and Tropical Medicine, London, UK; 5 INDEPTH Network, Accra, Ghana; 6 Department of Population Health, Faculty of Epidemiology and Population Health, London School of Hygiene and Tropical Medicine, London, UK

## Abstract

**Background:**

For people living with HIV (PLWH), the burden of travelling to a clinic outside of one’s home community in order to reduce the level of stigma experienced, may impact adherence to treatment and accelerate disease progression.

**Methods:**

This study is set in the Agincourt Health and Demographic Surveillance System (HDSS) in South Africa. Probabilistic and interactive methods were used to individually link HDSS data with medical records. A regression analysis was used to assess whether travel distance was correlated with the condition for which individuals were seeking care (primarily HIV, diabetes or hypertension). For PLWH, a Cox proportional hazard regression model was used to test for an association between the distance travelled to the clinic and late attendance at follow-up visits.

**Results:**

The adjusted relative risk (RR) of travelling to a clinic more than 5 km from that nearest to their home for HIV patients compared to those being treated for other conditions was 2.78 (95% confidence interval (CI) = 2.23-3.48). The adjusted Cox regression model showed no evidence for an association between the distance travelled to a clinic and the rate of late visits. (RR = 1.00, 95% CI = 0.99-1.00).

**Conclusions:**

The findings were consistent with the hypothesis that people living with HIV/AIDS would be willing to accept the burden of increased clinic travel distances in order to maintain anonymity and so limit their exposure to stigma from fellow community members. For those seeking HIV care the lack of an association between increased travel distances and late visit attendance suggests this may not impact treatment outcomes.

HIV prevalence amongst adults aged 15-49 was 18.9% in South Africa in 2016 (1). In that year, close to 4 million people were receiving anti-retroviral therapy (ART), this represented close to a 4-fold increase in the numbers on treatment since 2010 [1]. Of those commencing ART between 2004 and 2006 in South Africa 34.6% were no longer retained on treatment at 6 years after initiation [2]. Adequate adherence to antiretroviral therapy has been shown to be a critical determinant of successful treatment outcomes [3,4], hence it is important to understand factors associated with poor adherence as part of the response to the epidemic.

The accessibility of HIV care to those in need of treatment has been cited as likely to impact adherence, though evidence for an association between increased travel distances and poorer treatment outcomes is mixed [5], suggesting that the effect is context specific. Previous studies have shown evidence that people living with HIV/AIDS (PLWH) will travel beyond their nearest health care facility in order to access care [6,7]. A study carried out in a rural area of KwaZulu Natal in South Africa in 2010 showed that ART uptake was strongly negatively associated with the distance of an individual’s place of residence from the nearest clinic providing HIV treatment [8]. Another study in the same area from 2015 identified transport costs, which were associated with travel distance, and waiting times at the clinic, as important components of the overall economic burden for those seeking care for HIV and Tuberculosis (TB) [9]. This was a particularly significant burden for patients receiving ART due to the regularity of follow-up visits.

The factors driving an individual’s choice of where to seek HIV treatment are complex, driven by perceptions of the non-disease specific quality of care, barriers to access, costs [10] and expectations of encountering HIV-specific stigma from clinic staff and fellow community members [11,12].

In order to provide evidence to inform health systems planning we used linked clinical and demographic surveillance data to assess whether those being treated for HIV travelled further than those being treated for other conditions to seek care. We also investigated, amongst those being treated for HIV, whether those travelling further to the clinic were more likely to attend late for scheduled follow-up visits and hence be more likely to have poor treatment adherence.

To our knowledge this is the first study using data from a low and middle income country setting to specifically investigate whether, increases in the distance or time of travel to the clinic for those seeking treatment for HIV, lead to lower levels of adherence to treatment and poorer subsequent health outcomes.

## METHODS

**Figure 1:**
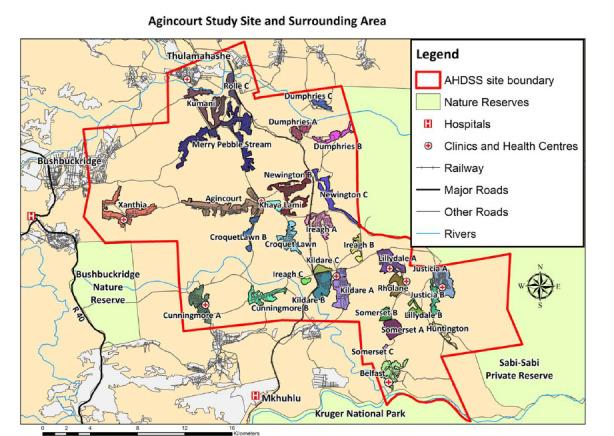
The location of the Agincourt HDSS in South Africa and the surrounding region.

This study was located in the Bushbuckridge sub-district of the Ehlanzeni municipality of Mpumalanga province, South Africa (**Figure 1**). This area hosts the Agincourt Health and Socio-Demographic Survey Site (AHDSS) [13].The site is mainly rural, though situated close to a number of peri-urban settlements. The area is characterised by having high levels of poverty and unemployment resulting in high levels of economic migration [14]. During the period of this study, 2014 to 2017, HIV testing and treatment services for AHDSS residents were available at seven public sector primary health care centres within the study site and at other clinics and hospitals beyond the study area. Individuals could present for HIV care at health facilities other than those closest to their place of residence, this allowed individuals to make evidence-informed choices on where to seek care.

All individuals presenting for chronic care at one of the seven clinics providing HIV care and treatment services in the study area between 3rd March 2014 and 14th August 2017, were screened for enrolment in the study. Follow-up data on subsequent visits was collected up to 19th Dec 2017.

Record linkage between clinic and AHDSS data was achieved using Point of Contact Interactive Record Linkage (PIRL) [15,16]. Conditional on informed consent, a dedicated data clerk obtained key identification data from each patient and then used a probabilistic algorithm to identify a set of possible matches with individuals in the AHDSS database. In consultation with the patient, the data clerk subsequently adjudicated between the different possible matches by checking the names of household co-residents that were retrieved from the AHDSS database. Linkage to the follow-up visit data was made using the patient identification number.

**Figure 2:**
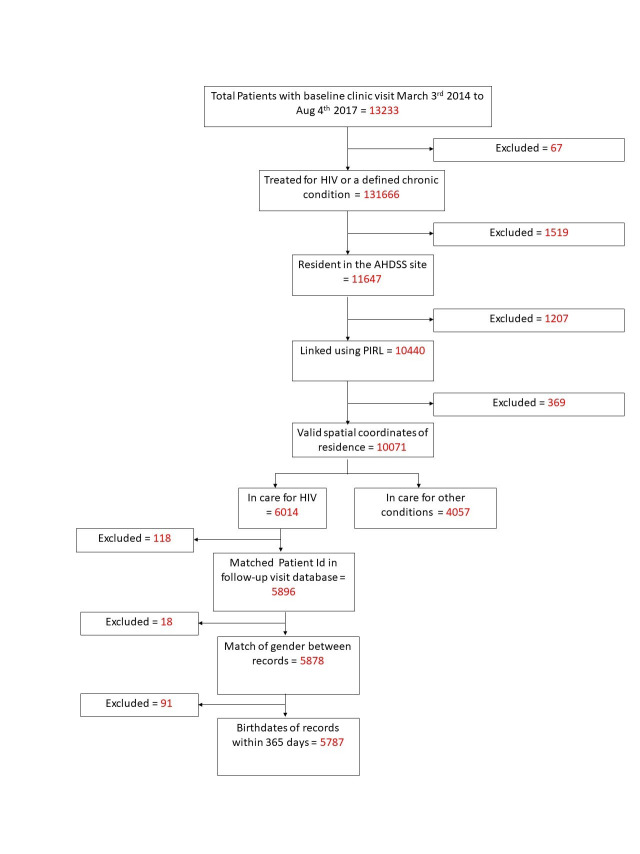
Flowchart showing the selection of the final study population.

Individuals were excluded if; they were not being treated for either HIV or a set of chronic conditions (hypertension tuberculosis, diabetes, asthma, epilepsy, mental illness, congestive cardiac failure and raised blood pressure), they reported being resident outside the study site, the PIRL linkage process failed to find a match with records in the AHDSS database or there was no record of the spatial coordinates of their place of residence. Additionally, individuals seeking care for HIV were excluded if when linking via the unique Patient Identifier number to the follow-up visit data set; there was no matching record, there was a mismatch of gender or the birthdates were more than 365 days apart. The numbers excluded due to each criteria are shown in the flowchart in **Figure 2**. All analyses were carried out using Stata version 14 [17].

The representativeness of the study population compared to all those seeking care for chronic conditions was assessed by comparing the characteristics of those included with those excluded.

The shortest distances by road from an individual’s place of residence to each clinic were calculated based on a network model of the major roads in the study site using the ArcGIS Network Analyst module [18]. Socio-economic Position (SEP) was defined based on a survey of household assets. Information was collected for 34 ordinal variables including features such as the materials used to construct the individuals dwelling, access to water and the ownership of appliances, livestock and transport. Based on this, households were assigned to SEP quintiles ranked from a value of 1, for the those with least assets, to 5, for those with the highest asset score [14]. Clinic names were anonymised to preserve confidentiality.

### Association between attending a local clinic and the presenting condition

For the initial analysis, the study outcome was a binary variable indicating whether or not the individual attended a clinic within 5 km by road of their place of residence. The primary exposure was a binary variable indicating whether the individual presented for treatment for HIV or another chronic condition. Modified Poisson regression analyses were used as the prevalence of the outcome exceeded 10% and log binomial models did not consistently converge [19]. Because individuals may have attended multiple clinics, we adjusted standard errors to account for the lack of independence between observations [20]. Explanatory variables which fulfilled conditions of theoretical and statistical plausibility to be considered as confounders were included in a multivariate model. A forward stepwise approach was used for the model building, at each step a Wald test was used out to test whether inclusion of the variable improved the model fit. The following variables were included in the analysis; age at first clinic visit, sex, clinic attended, year of first visit, country of origin (based on the individual’s father’s place of birth) and SEP. The inclusion of the clinic attended as a factor variable allowed us to consider the fixed effect of the clinic attended and whether this acted as an effect modifier. Following the creation of the initial multivariate model additional models were created including interaction terms between explanatory variables and the condition being treated. Wald tests were used to assess the strength of evidence for an interaction. Sensitivity analyses were carried out to investigate whether the study outcome was affected by the choice of cut-off distance.

### Association between the distance from home to the clinic by road and adherence to treatment

For the subset of individuals being treated for HIV, we used a Cox proportional hazard regression model to test whether there was an association between the distance travelled to the clinic and late attendance at follow-up visits. Late attendance was used as a proxy measure for poor adherence based on the assumption that individuals who attended visits late were likely to not have adequate supplies of ART to adhere to their prescribed treatment regimen. The period at risk was defined as the time from the first visit or the previous late visit, to the next late visit. A late visit was defined as an individual being more than 15 days late for an appointment and multiple failures were allowed for each individual. To adjust the standard errors to take account of the correlation of results, a variance covariance matrix in which individuals were clustered by their unique patient identifier code was used. Initially a series of bivariate models were built testing whether each explanatory variable was associated with the outcome. An additional categorical variable was included to indicate the length of time that an individual had been on ART at the first visit.

Variables were added to a multivariate model if there was either statistical or a priori evidence for their inclusion. A forward stepwise approach using Wald tests was used to select variables for inclusion in the multivariate model. The effect of interaction terms was also tested. For the final multivariate model, sensitivity analyses were carried out to test the effect of altering the cut-off used to define a late visit between 5 and 180 days.

### Ethics statement

This study received ethics approval from the University of Witwatersrand (#M141159) and the Mpumalanga Provincial Research and Ethics Committee.

## RESULTS

### Characteristics and representativeness of the study population

Of the 13 166 individuals who attended one of the seven study clinics for treatment for HIV or a chronic condition between 3 March 2014 and 14 August 2017, 3322 (25.2%) did not fulfil the inclusion criteria, leaving a total of 5787 (58.8%) 

seeking care for HIV and 4057 (41.2%) seeking care for other conditions in the final study population (**Figure 2**, Table S1 in the **Online Supplementary Document**). Of these 7411 (75.3%) were female, 7139 (72.5%) were of South African ethnic origin, 2685 (27.3%) of Mozambican origin and 20 (0.2%) had another unspecified ethnic origin (**Table1**).

**Table 1:** Results of bivariate and multivariate poisson regression analyses with an outcome attendance at a local clinic, defined as any clinic within 5 km by road of the nearest clinic to an individuals place of residence

		Travel to a clinic beyond 5 km from home for care			Risk ratio (95% CI)	
	**Category**	**No (%)**	**Yes (%)**	**Total**	**Bivariate (unadjusted) analysis**	**Multivariate (adjusted) analysis ^1^**
Condition	Other	3296(96.8)	131(3.2)	4057	1	1
	HIV	5054(87.3)	733(12.7)	5787	3.92 (3.27-4.70)	2.78 (2.23-3.48)
Age at first clinic visit	17-34	2527 (86.6)	391 (13.4)	2918	1	1
	35-49	2546 (89.6)	296 (10.4)	2842	0.78 (0.68-0.89)	0.95 (0.81-1.11)
	50-64	2100 (94.8)	115 (5.2)	2215	0.39 (0.32-0.47)	0.65 (0.51-0.81)
	>65	1805 (96.7)	62 (3.3)	1867	0.25 (0.19-0.32)	0.58 (0.42-0.80)
Sex	Female	6730 (90.8)	681 (9.2)	7411	1	1
	Male	2250 (92.5)	183 (7.5)	2433	0.82 (0.70-0.96)	0.79 (0.66-0.94)
Health Facility attended	Arlington	1609 (97.4)	43 (2.6)	1652	1	1
	Faith	1071 (84.1)	203 (15.9)	1274	6.12 (4.45-8.43)	4.71 (3.34-6.65)
	Hillard	714 (87.3)	104 (12.7)	818	4.88 (3.46-6.90)	5.97 (4.10-8.70)
	Moghan	1077 (95.6)	50 (4.4)	1127	1.70 (1.14-2.54)	1.80 (1.17-2.78)
	Timber	953 (96.5)	35 (3.5)	988	1.36 (0.88-2.11)	1.16 (0.72-1.85)
	Troy	2448 (86.6)	380 (13.4)	2828	5.16 (3.79-7.04)	4.66 (3.33-6.53)
	Yang	1108 (95.8)	49 (4.2)	1157	1.63 (1.09-2.43)	1.65 (1.06-2.56)
Socio Economic Position	1	1332 (92.6)	107 (7.4)	1439	1	1
	2	1595 (92.6)	127 (7.4)	1722	0.99 (0.78-1.27)	1.02 (0.80-1.29)
	3	1600 (91.7)	144 (8.3)	1744	1.11 (0.87-1.41)	1.13 (0.89-1.42)
	4	1755 (91.2)	170 (8.8)	1925	1.19 (0.94-1.49)	1.31 (1.04-1.64)
	5	1560 (90.6)	161 (9.4)	1721	1.26 (1.00-1.59)	1.49 (1.18-1.88)
Ethnic origin	Mozambican	2415 (89.9)	270 (10.1)	2685	1	1
	South African	6552 (91.8)	587 (8.2)	7139	0.82 (0.71-0.94)	0.65 (0.55-0.77)
	Other	13 (65.0)	7 (35.0)	20	3.48 (1.95-6.20)	2.26 (1.09-4.66)
Year of first clinic visit	2014	5586 (93.4)	392 (6.6)	5978	1	1
	2015	1421 (88.2)	191 (11.8)	1612	1.81 (1.53-2.13)	1.28 (1.07-1.53)
	2016	1388 (88.4)	182 (11.6)	1570	1.77 (1.50-2.09)	1.28 (1.06-1.54)
	2017	585 (85.5)	99 (14.5)	684	2.21 (1.80-2.71)	1.62 (1.29-2.04)
	**Total**	8980 (91.2)	864 (8.8)	9844		

CI – confidence interval

*Adjusted for all variables shown.

When the characteristics of those excluded were compared with those for the final study population (Table S1 in the **Online Supplementary Document**). it was found that the following groups were more likely to be excluded; those seeking care for HIV compared to those seeking care for other conditions (30.3% v 16.7%), males compared to females (26.5% vs 24.8%) and those of Mozambican origin compared to South Africans (13.6% vs 10.1%). The percentage excluded decreased with increasing age from 30.9% for those aged 17 to 34 to 14.8% for those aged greater than 65. There was an increase in the percentage excluded for each successive year of recruitment from 20.6% in 2014 to 36.5% in 2017. There was also considerable variation in the percentage excluded by clinic, ranging from 13.1% of those attending to 47.2% of those attending Moghan.

Of the 4507 individuals presenting with conditions other than HIV, 3648 (89.9%) were being treated for hypertension, 637 (15.7%) for diabetes, 274 (6.8%) for mental illness, 177 (4.4%) for epilepsy, 135 (3.3%) for asthma, 92 (2.3%) for tuberculosis and 22 (0.5%) for congestive cardiac failure (Table S2 in the **Online Supplementary Document**).

### Risk of not seeking care at the nearest clinic

The unadjusted bivariate analysis showed that the risk ratio (RR) for HIV patients compared to those being treated for other conditions of travelling to a clinic more than 5 km from that nearest to their home was 3.92 (95% CI = 3.27-4.70) (**Table 1**). This analysis also showed that the RR decreased with increasing age, the RR for those aged 35 to 49 compared to those aged 17 to 34 was 0.78 (95% CI = 0.68-0.89) whilst for those aged over 65 the RR was 0.25 (95% CI = 0.19-0.32). Males had a lower risk of travelling to a distant clinic than females (RR = 0.83, 95% CI = 0.70-0.96). Those of South African ethnic origin had a lower risk of travelling to a distant clinic than those who were ethnically Mozambican (RR = 0.82, 95% CI = 0.67-0.95). There was also significant variation depending on the clinic attended. There was limited evidence that the risk varied according to an individual’s socio-economic position or by the year they first visited the clinic. After adjusting for the effect of other variables in a multivariate model the RR decreased to 2.78 (95% CI = 2.23-3.48) (**Table 1**).

Adding an interaction term to the multivariate model provided no statistical evidence that the association between attending a non-local clinic and the condition being treated differed according to which health facility an individual attended (*P*-value for the Wald test on the interaction term = 0.38).

The sensitivity analysis to test the effect of different cut-off distances to define a local clinic (Table S3 in the **Online Supplementary Document**) indicated that the adjusted RR increased from 2.01 (95% CI = 1.64-2.46) when the cut-off was 0 km to 3.90 (95% CI = 2.08-7.29) when a 7 km cut-off was used.

### Association between the distance travelled to the clinic by road and the rate of late visits

In this analysis the rate of late visits was used as a proxy measure of poor adherence to treatment. There were 12428 instances of an individual attending more than 15 days late for a follow-up appointment during a total of 16585 years of follow-up, a rate of 0.75 events/person year (**Table 2**). In the unadjusted (bivariate) analysis there was no evidence that the rate of late visits increased with increasing distance from home to the health facility, hazard ratio (HR) = 1.00 (95% CI = 1.00-1.01). The rate of late visit attendance decreased with increasing age, HR for those aged 50 to 64 compared to those aged 17 to 34 was 0.69 (95% CI = 0.65-0.74). Males had a higher rate of late attendance than females (HR = 1.16, 95% CI = 1.11-1.23). The rate increased with each subsequent year of first recorded clinic visit, the HR for those first visiting in 2017 compared to 2014 was 1.48 (95% CI = 1.35-1.63).

**Table 2:** The relationship between the rate of attendance for clinic visits and the distance travelled – the results of a a Cox regression multiple failure model.

Variable	Category	Events/Person years of observation	Event Rate	Hazard ratio* (95% confidence interval) (Bivariate analysis)	Hazard ratio*,† (95% confidence interval), (Multivariate adjusted analysis)
**Distance travelled from home to clinic (km)**		12 428/16 585	0.75	1.00 (1.00-1.01)	1.00 (0.99-1.00)
**Age at first clinic visit**	17-34	5589/6608	0.85	1	1
	35-49	4601/6315	0.73	0.84 (0.80-0.89)	0.84 (0.80-0.88)
	50-64	1779/2938	0.61	0.69 (0.65-0.74)	0.70 (0.65-0.75)
	>65	459/725	0.63	0.72 (0.63-0.83)	0.74 (0.64-0.84)
**Sex**	Female	9253/12 800	0.72	1	1
	Male	3175/3785	0.84	1.16 (1.11-1.23)	1.24 (1.18-1.30)
**Health Facility attended for baseline visit**	Arlington	1543/2514	0.61	1	1
	Faith	1955/2273	0.86	1.42 (1.30-1.55)	1.41 (1.29-1.54)
	Hillard	772/1157	0.67	1.05 (0.92-1.20)	1.06 (0.93-1.20)
	Moghan	827/1811	0.46	0.73 (0.65-0.82)	0.76 (0.67-0.85)
	Timber	1568/1913	0.82	1.32 (1.20-1.45)	1.34 (1.22-1.47)
	Troy	4304/5268	0.82	1.34 (1.25-1.45)	1.36 (1.26-1.46)
	Yang	1459/1650	0.88	1.43 (1.31-1.57)	1.46 (1.34-1.60)
**Socio Economic Position**	1	1963/2773	0.71	1	
	2	2276/3043	0.75	1.06 (0.98-1.14)	
	3	2357/3091	0.76	1.08 (1.00-1.17)	
	4	2212/2946	0.75	1.07 (0.99-1.16)	
	5	1659/2322	0.71	1.02 (0.94-1.10)	
**Ethnic origin**	Mozambican	3621/4882	0.74	1	1
	South African	8776/11 662	0.75	1.02 (0.97-1.07)	1.06 (1.01-1.12)
	Other	31/41	0.76	1.02 (0.62-1.70)	1.02 (0.68-1.54)
**Year of first clinic visit**	2014	7945/11 147	0.71	1	1
	2015	2326/2816	0.83	1.27 (1.20-1.35)	1.21 (1.14-1.28)
	2016	1639/2002	0.82	1.34 (1.26-1.43)	1.28 (1.20-1.36)
	2017	518/620	0.84	1.48 (1.35-1.63)	1.41 (1.28-1.54)
**Time on ART at baseline (years)**	0	2565/3166	0.81	1	
	>0–1	3466/4423	0.78	0.94 (0.88-1.00)	
	>1-2	1700/2333	0.73	0.84 (0.78-0.91)	
	>2-3	1936/2780	0.70	0.80 (0.74-0.86)	
	>3	2761/3884	0.71	0.83 (0.78-0.89)	

CI – confidence interval

*Failure is defined as a visit later than 15 days after the due date and the period of exposure was the time from the first visit or the previous late visit.

†Adjusted for all variables shown.

In the multivariate model adjusting for age, sex, health facility attended, ethnic origin and year of first clinic visit there remained no evidence for an association between the distance travelled to a health facility and the rate of late visits. (HR = 1.00, 95% CI = 0.99-1.00). In a sensitivity analysis the cut-off used to define a late visit was adjusted in increments between 5 and 180 days after the due date for the visit (Table S4 in the **Online Supplementary Document**), the rate ratios obtained were indistinguishable from those obtained using the 15-day cut-off in our primary model (**Table 2**).

In a further model an interaction term between health facility attended the distance travelled was added and stratum specific rate ratios were calculated. (**Table 3**). Evidence for an association between the distance travelled to a health facility and the rate of late visits was only seen for Arlington clinic (HR = 1.02, 95% CI = 1.01-1.04).

**Table 3:** The relationship between the rate of attendance for clinic visits and the distance travelled for each individual clinic

Clinic attended	Hazard ratio* (95% CI)
Arlington	1.02 (1.01-1.04)
Faith	0.99 (0.98-1.00)
Hillard	0.99 (0.98-1.00)
Moghan	0.99 (0.97-1.01)
Timber	0.99 (0.97-1.01)
Troy	1.00 (0.99-1.01)
Yang	1.01 (0.99-1.02)

CI – confidence interval

*The Cox regression multiple failure model includes a term representing the interaction between clinic attended and distance travelled to attend that clinic. Failure is defined as a visit later than 15 days after the due date and the period of exposure was the time from the first visit or the previous late visit.

## DISCUSSION

In this study we found strong evidence that those with HIV were willing to travel further than those with other conditions to seek care. Those seeking care for HIV were almost four times more likely to travel to a distant clinic, defined as one more than 5 km from the nearest clinic to home, than those being treated for other conditions. After controlling for the effect of other confounding factors the relative risk reduced to just less than three. We also found that those who were younger, female, of Mozambican ethnic origin, and who had first been enrolled after 2014, were more likely to travel to a distant clinic to seek care. We found no evidence for an association between the rate of late attendance for clinic visits, used as a proxy for poor treatment adherence, and the travel distance to the clinic. We did however find that the rate of late attendance for follow-up visits was greater for those who; were of younger age, were male, had first been enrolled after 2014 and who had not started antiretroviral treatment at enrolment.

A similar effect was seen in a study in Uganda in which PLWH travelled greater distances to access care than those not living with HIV. This was largely based on lack of provision of ART at their nearest clinic, although there was some evidence that individuals had a preference for clinics providing a higher grade service [6]. A study in a rural community in Uganda showed that 57% of those seeking care for HIV travelled further than their nearest clinic and that those with higher levels of education and higher wealth were more likely to travel further [7]. A study of patterns of travel for HIV care in the UK found that 25% of patients chose to attend a non-local service, defined as a clinic more than 5 km from their nearest clinic [21]. They also showed that the use of non-local services was twice as common for residents of the least deprived areas as it was for residents of the most deprived areas.

As HIV remains a highly stigmatised disease [11,12], one factor leading to individuals choosing to travel further than their closest clinic for HIV care may be a desire to lessen the likelihood of encountering fellow community members during the visit. Perceptions of a higher quality of HIV care from a more distant clinic may also contribute.The three A’s framework is a useful theoretical model for understanding the interplay of different barriers associated with accessing health care [10]. In this framework, access is conceptualised as having three dimensions; **availability** –the timeliness and geographical accessibility of care, **affordability** – financial costs associated with care and **acceptability** – the degree to which cultural factors and personal choice based on the perceived quality of care influences health care seeking behaviour. As HIV care is proved free of charge in this setting, these results suggest that individuals are willing to sacrifice ‘availability’ in order to seek more ‘acceptable’ care for HIV.

The lack of evidence for an association between travel distance and HIV treatment attendance suggests that interventions which make travel to clinic easier such as building new clinics or subsidising travel costs would not be expected to improve treatment outcomes in this setting. By comparison a systematic review investigating the association between geographic and transportation barriers and HIV care outcomes in sub-Saharan Africa countries found 23 studies in which increased travel distances led to worse care outcomes, 26 in which increased travel distances had no effect and 3 in which additional distances had a beneficial effect [5]. This led the authors of that study to conclude that interventions which addressed geographical barriers to accessing care could lead to higher levels of engagement and retention through the HIV care continuum. Further evidence from more recent studies in China [22], Zimbabwe [23], the USA [24] and Canada [25] would support this conclusion.

The lack of an association between SEP and either differential travel distance or adherence was of interest. Previous studies in this community [26] carried out between 2007 and 2010 indicated that higher levels of SEP, indicative of greater material wealth, were associated with lower levels of HIV related mortality. Other authors have suggested that the association between SEP and risk of acquiring HIV may change over time [27], which may explain our finding.

Both the rate of non-adherence to follow-up visits and the relative likelihood of travelling to a non-local clinic for those with HIV increased between 2014 and 2015 and subsequently stayed at similar levels. Further study is needed to understand whether any changes in practice took place at the clinics between those years.

There were several limitations to this study. The final study population, as compared to all eligible clinic attendees, contained a lower percentage of persons of younger age, Mozambican origin, and of those enrolled in later years of the study, this may limit the generalisability of our findings. As biological markers of adherence such as CD4 count, viral load or levels of ART drugs in the blood were not available, we used the rate of late attendance at follow-up visits was used as a proxy measure.

A major strength of this study is the availability of linked data on individuals’ sociodemographic characteristics and their clinical history which enabled us to explore the interplay between different factors contributing to the choices individual’s make about where to seek HIV care. Also as this study was carried out in a high HIV burden low and middle income country, the findings can inform efforts to optimise the provision of HIV care in such settings.

## CONCLUSIONS

In summary this study adds to evidence that patterns of health seeking behaviour differ for those with highly stigmatised conditions such as HIV compared to those treated for other conditions. Our results indicate that in this setting the perceived advantage of experiencing more acceptable care for HIV by travelling to a distant clinic outweighs the inconvenience of longer treatment-seeking journeys. Hence specifically in the Bushbuckridge sub-district, interventions to reduce clinic distances are likely to be less effective than other measures in improving the overall quality of HIV care. We would further recommend that health planners consider the adoption of this analytical approach in other places in order to gain a better understanding of local patterns of health seeking behaviour and to identify clinics providing care that is less acceptable to local populations.

## Additional material

Online Supplementary Document
