# Supplementary material for: The impact of HIV status on the distance traveled to health facilities and adherence to care. A record-linkage study from rural South Africa
Source: J Glob Health. 2020 Dec 6;10(2):020435. doi: 10.7189/jogh.10.020435 (PMC7719896; doi:10.7189/jogh.10.020435)
Supplement: Online Supplementary Document [file jogh-10-020435-s001.pdf]

Supplementary Materials

		Included in the final study population		
		**No (%)**	**Yes [1] (%)**	**Chi-squared p-value**
**Condition**	**Other**	811 (16.7)	4057 (83.3)	< 0.01
	**HIV**	2511 (30.3)	5787 (69.7)	
**Sex**	**Female**	2446 (24.8)	7413 (75.2)	0.05
	**Male**	876 (26.5)	2431 (73.5)	
**Age at first visit**	**17-34**	1308 (30.9)	2926 (69.1)	< 0.01
	**35-49**	1108 (28.0)	2845 (72.0)	
	**50-64**	514 (19.2)	2168 (80.8)	
	**>65**	314 (14.8)	1812 (85.2)	
**Year of first visit**	**2014**	1550 (20.6)	5978 (79.4)	< 0.01
	**2015**	632 (28.2)	1612 (71.8)	
	**2016**	746 (32.2)	1570 (67.8)	
	**2017**	394 (36.5)	684 (63.5)	
**Ethnic Origin**	**Mozambican**	421 (13.6)	2685 (86.4)	< 0.01
	**South African**	800 (10.1)	7139 (89.9)	
	**Other**	2101 (99.1)	20 (0.9)	
**Health Facility attended**	**Arlington**	249 (13.1)	1652 (86.9)	< 0.01
	**Faith**	558 (30.5)	1274 (69.5)	
	**Hillard**	482 (37.1)	818 (62.9)	
	**Moghan**	1007 (47.2)	1127 (52.8)	
	**Timber**	212 (17.7)	988 (82.3)	
	**Troy**	591 (17.3)	2828 (82.7)	
	**Yang**	223 (16.2)	1157 (83.8)	
	**Total**	**3322 (25.2)**	**9844 (74.8)**	

Table S1 A comparison of the percentages of each strata excluded and included in the final study population for various characteristics of the study population.

[1] For unlinked patients only data recorded in the clinic is available, hence clinic data is used for comparison purposes in this table. In table 1 values recorded in the AHDSS database were used. As a result there are minor numerical differences in the strata totals for age and sex between this table and table 1.

Presenting Condition	Number treated for the condition (Percentage of all individuals)
Hypertension	3648(89.9)
Diabetes	637(15.7)
Mental Illness	274(6.8)
Epilepsy	177(4.4)
Asthma	135(3.3)
Tuberculosis	92(2.3)
Congestive Cardiac Failure	22(0.5)
**Total Individuals**	**4057**

Table S2 Presenting conditions for individuals being treated for a range of chronic conditions other than HIV. Individuals could receive treatment for multiple conditions.

Cut off distance used to define local [2] clinic (km)	Multivariate adjusted risk ratio [1] (95% Confidence interval)
0	2.01 (1.64-2.46)
3	2.48 (1.94 – 3.68)
5 *	2.96 (2.01 – 4.34)
7	3.90 (2.08 – 7.29)

Table S3 - Multivariate adjusted risk ratios comparing the risk of attending a local clinic with that for attending one that is not local, using a range of cut-off distances for the excess distance travelled to the clinic that is not the nearest by road to an individual's place of residence.

[1] Derived from a multivariate model in which the association between being an HIV client and attending a clinic within the indicated distance of home was adjusted for age at first visit , sex, mean gaps between visits, health facility attended, nearest health facility, socio-economic position and the interaction between being an HIV client and the variable shown in each respective table
[2] A local clinic is defined as one within the indicated distance by road of the nearest clinic.
* The cut off of 5 km is that included in the multivariate models presented in table 3 and is included here for the purpose of comparison.

Cut-off used to define a late visit (Days)	Multivariate [1] analysis Hazard Ratio (95% CI) Effect of distance travelled from home to clinic (km)
5	1.00 (1.00-1.01)
10	1.00 (1.00-1.01)
15	1.00 (0.99-1.00)
20	1.00 (1.00-1.00)
25	1.00 (0.99-1.00)
30	1.00 (0.99-1.00)
90	1.00 (0.99-1.01)
180	0.98 (0.97-1.00)

Table S4 – Sensitivity analysis to evaluates the effect of choosing different definitions of the cut-off time used to define a late visit in multivariate Cox regression models assessing the impact of increased distances travelled from home to the clinic on the rate of late visits the model allows for multiple failures (late visits) for each individual. The multivariate model we adjusted for the age at first clinic visit, sex, health facility attended, ethnic origin and year of first visit.
